# The Human Proteoform Project: Defining the human proteome

**DOI:** 10.1126/sciadv.abk0734

**Published:** 2021-11-12

**Authors:** Lloyd M. Smith, Jeffrey N. Agar, Julia Chamot-Rooke, Paul O. Danis, Ying Ge, Joseph A. Loo, Ljiljana Paša-Tolić, Yury O. Tsybin, Neil L. Kelleher, The Consortium for Top-Down Proteomics

**Affiliations:** 1 Department of Chemistry, University of Wisconsin, Madison, WI, USA.; 2 Departments of Chemistry and Chemical Biology and Pharmaceutical Sciences, Northeastern University, Boston, MA, USA.; 3 Department of Structural Biology and Chemistry, Institut Pasteur, CNRS, Paris, France.; 4 Consortium for Top-Down Proteomics, Cambridge, MA, USA.; 5 Department of Cell and Regenerative Biology, Department of Chemistry, Human Proteomics Program, University of Wisconsin-Madison, Madison, WI, USA.; 6 Department of Chemistry and Biochemistry, University of California, Los Angeles, Los Angeles, CA, USA.; 7 Pacific Northwest National Laboratory, Richland, WA, USA.; 8 Spectroswiss, Lausanne, Switzerland.; 9 Departments of Chemistry, Molecular Biosciences and the Feinberg School of Medicine, Northwestern University, Evanston, IL, USA.

## Abstract

Proteins are the primary effectors of function in biology, and thus, complete knowledge of their structure and properties is fundamental to deciphering function in basic and translational research. The chemical diversity of proteins is expressed in their many proteoforms, which result from combinations of genetic polymorphisms, RNA splice variants, and posttranslational modifications. This knowledge is foundational for the biological complexes and networks that control biology yet remains largely unknown. We propose here an ambitious initiative to define the human proteome, that is, to generate a definitive reference set of the proteoforms produced from the genome. Several examples of the power and importance of proteoform-level knowledge in disease-based research are presented along with a call for improved technologies in a two-pronged strategy to the Human Proteoform Project.

## Teaser

An ambitious initiative is proposed to generate a definitive reference set of human proteoforms.

The Human Genome Project (HGP) was a remarkable and unqualified success profoundly transforming and accelerating biological and medical research while converting a ~ $4B public investment into over $700B of economic activity and new industries (*1*). The challenge of revealing the “Blueprints of Life,” however, is surpassed by the challenge we face today: deriving from these blueprints an understanding of the structures they dictate and how these function within biological systems.

**Fig. 1.**
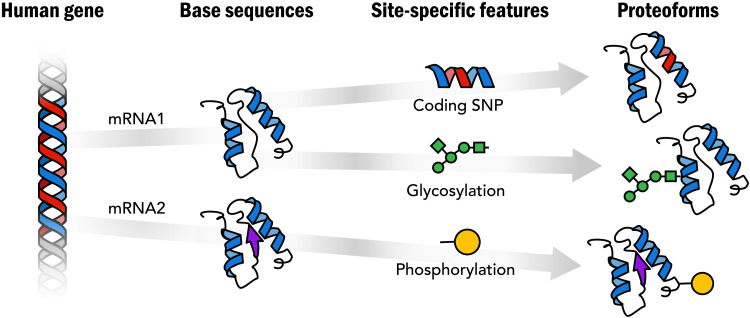
Proteoforms: Distinct protein forms arising from a single gene.

Proteins are primary effectors of function in biology, and thus, complete knowledge of their structure and behavior is fundamental to deciphering function in basic and translational research (*2*). The richness of protein structure and function goes far beyond the linear amino acid sequence dictated by the genetic code. Genetic variation, alternative splicing, and posttranslational modification (PTM) work together to create a rich variety of different proteoforms arising from our genes (Fig. 1) (*3*). The chemical diversity of proteins is foundational for the biological complexes and networks that control biology yet remains largely unknown. Genome sequence alone does not provide the needed information—only direct analysis of the proteoforms themselves can reveal their composition, enabling studies of their spatial distributions and temporal dynamics in biological systems. We propose here an ambitious initiative to define the human proteome, that is, to generate a definitive set of reference proteoforms produced from the genome (see Box 1).

Box 1. What is a proteome?

A standard answer to this question is that a proteome is the set of proteins expressed by an organism. This idea clearly depends on what is meant by a “protein.” Proteins from even a single gene can vary widely in their amino acid sequence and PTMs giving rise to a variety of proteoforms. Thus, the proteome is necessarily the set of all proteoforms expressed by an organism. The initiative proposed here is founded upon this simple idea.

## PROTEOFORM-LEVEL KNOWLEDGE IS ESSENTIAL TO UNDERSTAND BIOLOGICAL FUNCTION

Proteins are the central intermediaries between genotype and phenotype (*2*–*4*). It is not possible to understand the functioning of a biological system if one does not know what protein molecules are present, as well as the nature and abundances of their proteoforms. Knowledge of where the proteoforms are located within cells or tissues, what other proteoforms they interact with to form the multifunctional complexes that carry out critical functions in cell biology, and how they change in response to stimuli is essential. Innovative new tools are needed to comprehensively define the proteome, allowing proteoform abundances, interactors, and locations to be assessed with far greater depth at lower cost. The foundational premise of the HGP, which knowledge of the genome sequence will provide a fundamental understanding of biological systems, will not be realized in the absence of detailed proteoform-level information. This was clearly articulated by Collins *et al*. (*2*), “A critical step toward gaining a complete understanding... will be to take an accurate census of the proteins present in particular cell types. It will be a major challenge to catalog proteins present in low abundance or in membranes. Determining the absolute abundance of each protein, including all modified forms, will be an important next step.”

**Fig. 2.**
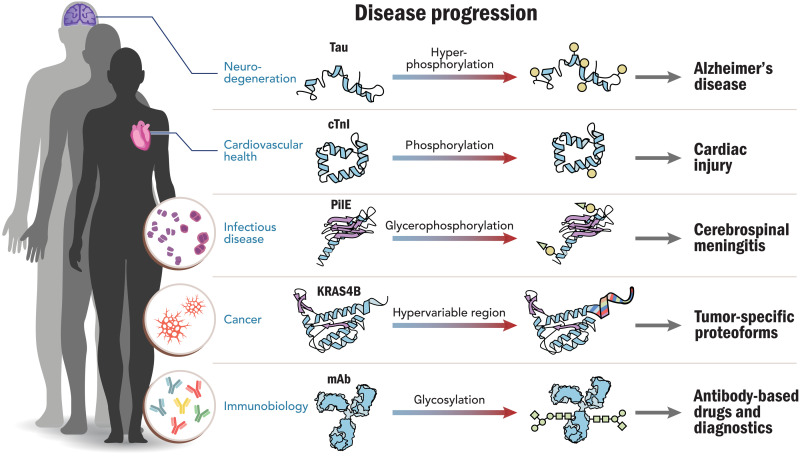
Proteoforms in human disease. Five important clinical areas of interest are depicted and serve as examples where proteoforms have been identified and linked to the progression of human disease; they are discussed at length in an extended preprint version of this *Perspective* (*32*). mAb, monoclonal antibody.

The Human Proteoform Project we present here is the critical next step in the quest to understand human health and disease. Several examples from five important disease areas illustrate the critical role of proteoforms in disease and health (Fig. 2). These examples show how disease-driven research has been advanced by discovery of proteoforms and their PTMs.

## CENTRAL GOALS AND STRATEGY OF THE PROJECT

The primary objective of this project is to elucidate a complete set of expressed proteoforms derived from the ~20,000 genes encoded in the human genome. We forward a two-pronged strategy: On the one hand, we pursue deep proteoform-level analysis in medically relevant systems (Fig. 2); this will continue to open up fundamental insights into targets and use cases of high biomedical importance. In parallel, we invest heavily in the accelerated development of proteoform discovery and characterization technologies and deploy them for large-scale proteoform analysis to specimens from nominally healthy donors.

The project is modeled roughly after the successful roadmap provided by the HGP, which generated the human reference genome sequence while advancing technology in the process (*2*, *3*, *5*). An international effort on the scale of the HGP in both funding and time will reveal the full chemical complexity of our proteins, drive the frontiers of research and medicine well beyond what is currently possible, and be critical in the assignment of function to proteins and their PTMs in the decades ahead.

## THE HUMAN PROTEOFORM PROJECT

We propose the Human Proteoform Project, a program to aggressively develop new technologies for comprehensive proteoform analysis and to assemble an extensive, high-quality atlas of human proteoforms. We envision next-generation proteomics in humans to be based on ~20,000 proteoform families (*6*), one for each gene in the genome. Deep catalogs of proteoforms compiled for widely characterized mammalian cell lines and primarily human samples will markedly accelerate our understanding and exploitation of proteins. This more profound knowledge of the central molecules of biology will provide an essential cornerstone for 21st century biology. New technologies will be central to this effort, as today’s ability to comprehensively identify proteoforms in complex systems is limited.

## ASSEMBLING THE HUMAN PROTEOFORM ATLAS

**Fig. 3.**
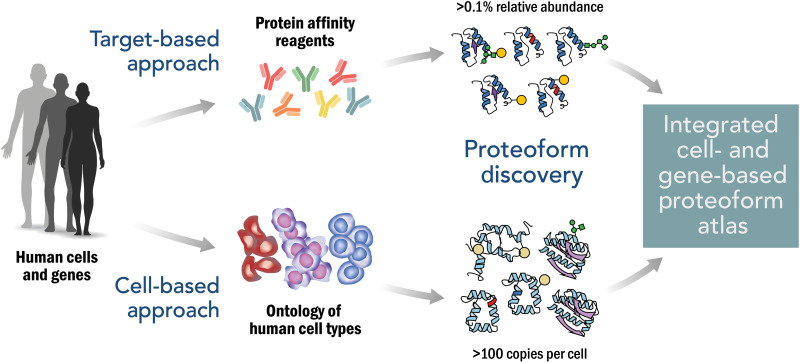
Approach to creating an integrated Human Proteoform Atlas. The upper path illustrates the use of protein affinity reagents to capture proteoform families derived from targeted genes. The lower path illustrates the in-depth analysis of human cell types for proteoform discovery and characterization. Relative abundance refers to the ratio of a given proteoform to the sum of all proteoforms in that family.

Proteoform expression varies across cells and tissues, and studies of proteoform expression can be either global or targeted. The expression of rare proteoforms is stochastic in nature. The Human Proteoform Project will thus necessarily focus on capturing the identities of the dominant functional proteoform population rather than rare occurrences. We propose the bifurcated approach shown in Fig. 3. In global studies, all proteoforms present at detectable levels are characterized; in targeted studies, specific proteoform families arising from each human gene will be enriched and subjected to systematic proteoform discovery to reveal the molecular diversity present. The two paths are described below.

### Cell-based approach to proteoform discovery

An important thrust of the project is the delineation of proteoform expression patterns in human cell types (Fig. 3, bottom) (*7*). Defining the number and nature of human cell types is an ambitious undertaking in its own right and is currently being pursued by several consortia (see below). Anchoring proteoform analysis with cell types provides a generalized strategy to access human biology across the natural context present within our tissues. The depth of proteoform analysis obtained depends on the detection sensitivity of the technology used: While today’s mass spectrometric platforms are pushing toward detection limits of ~25 copies per cell (*7*), aggressive technology investment is needed to further develop these platforms and to develop new approaches and paradigms (see the section below). A cell-based approach can begin using many thousands of cells of a given type and adopt single-cell proteoform technologies as they become available.

### Gene-based approach for targeted proteoform discovery

**Fig. 4.**
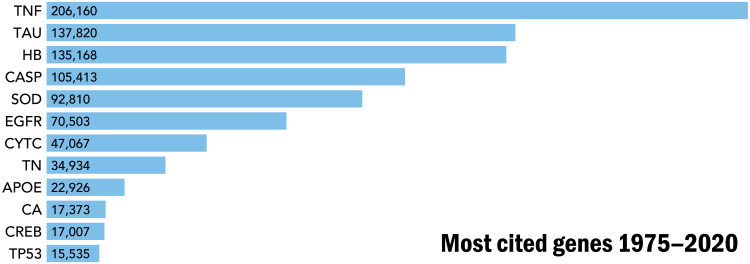
The most studied proteins have essential proteoforms that contain common PTMs such as phosphorylation, methylation, acetylation, and other important variations of primary structure such as disulfide bond formation, metal attachment, and proteolytic processing. TNF, tumor necrosis factor; TAU, tubulin-associated unit; HB, hemoglobin subunits (HbA, HbB, etc.); CASP, cysteine-aspartic proteases (Casp1 to Casp9); SOD, superoxide dismutases (SOD-1, SOD-2, and SOD-3); EGFR, estrogen growth factor receptor; CYTC, cytochrome C; TN, troponin (Tn-C, Tn-I, and Tn-T); APOE, apolipoprotein E; CA, carbonic anhydrase; CREB, cyclic adenosine 5′-monophosphate response element–binding protein; TP53, cellular tumor antigen p53. Citations are from the Web of Science Core Collection from 1975 to 2020. Citations per year and a history of research trends have been chronicled for a subset of these proteins (*46*).

The development of affinity reagents to capture the proteins encoded by each human gene will be invaluable to enrich and then characterize their proteoform families in a selection of human specimens. The fundamental role of proteoform-level knowledge in understanding human disease and health (Fig. 2) is evident from consideration of the most highly cited human genes in the biomedical literature (Fig. 4). Tumor necrosis factor, at the top of the list, has >200,000 citations; this high-citation number can be considered a reasonable proxy for the research funding that has gone into its study over decades. Notably, even the most-studied genes have unknown proteoforms essential to understand of their biological and disease-related functions. The economies of scale afforded by a concerted project to obtain comprehensive proteoform-level knowledge will make possible the acquisition of such information for the 20,000 proteoform families derived from the human genome.

## NEW TECHNOLOGIES

At present, the dominant “bottom-up” paradigm of mass spectrometry (MS)–based proteomics sacrifices information about proteoforms by cleaving proteins into peptides; this is done for a pragmatic reason—it works, as the resultant peptides are generally much easier to identify than their parent intact proteoforms (*8*, *9*). Top-down proteomics, in contrast, analyzes the entire intact proteoform and is the most powerful proteoform-level analysis technology in existence, providing knowledge regarding RNA isoform translation and combinatorial PTMs, but is limited in depth and throughput (*4*, *6*). The flagship efforts of the Cancer Proteomics Consortium, CPTAC, have brought targeted proteomics and proteogenomics into regular use and produced major studies on ovarian (*10*), breast (*11*), and colorectal cancer (*12*). Using the bottom-up approach to proteomics, CPTAC noted recently that “the aggregated NCI-60 proteomics dataset covers only 12% of the whole encoded proteome, and only ~5% of the genes had sequence coverage of >50% of their protein coding regions.” (*13*). Regarding alternative splicing, “there is yet a major gap between the number of alternative transcripts asserted by RNA sequencing and that detectable by proteomics (e.g., <0.1% of putative novel splice junctions in cancer xenografts)” (*13*). This state of affairs underscores the critical need to advance the state of the art in proteomic analysis (*14*–*17*) via new technologies and extensive proteoform–level characterization of biological systems.

To achieve the objectives outlined above, it is critical to expand our technological abilities through a concerted long-term and multifaceted research and development effort. This effort should pursue both the continued development of MS-based technologies for proteoform analysis, as well as the exploration of potential paradigm-shifting new ideas and approaches that offer the possibility of transformative change. The development of increasingly powerful and effective nucleic acid sequencing has demonstrated the importance of investing heavily in ambitious new efforts to drive technology development. Similarly, single-molecule MS (*18*–*20*), nanopore sequencing (*21*, *22*), cryoelectron microscopy and visual proteomics (*23*, *24*), single-cell proteomics (*25*–*29*), single-molecule protein arrays (*30*, *31*), and other ideas yet to be conceived need to be encouraged, supported, and developed to advance proteoform biology.

The outstanding success of the technology development program in the HGP and the associated private sector engagement provide an inspiring model for how this can be done well. Just as the $1 per base estimate for the HGP provided an important target to spur technology competition and development, so will a $1 per proteoform goal for the Human Proteoform Project as proposed previously (*7*). Although the details of its implementation plan will be developed with key stakeholders, at this time, the main parameters and their estimates help frame the project. For the cell-based prong (Fig. 3), we can anticipate that the output of the Human Cell Atlas, Human Biomolecular Atlas Program (HuBMAP), and other consortia will be a defined ontology and number of human cell types, allowing the proteome of each to be targeted. Assuming 5000 cell types and prescribing a depth of 1 million proteoforms in each, constructing the Human Proteoform Atlas would involve ~5 billion measurements of redundant proteoforms (*32*). Combined with the gene-based approach, perhaps ~50 million unique (nonredundant) proteoforms will be asserted with defined quality metrics over the course of the project.

## THE PIVOT FROM PROTEOFORM DISCOVERY TO PROTEOFORM SCORING

A central principle in comprehensive proteoform analysis concerns the distinction between discovery and scoring. Comprehensive analysis of protein primary structure requires the generation of highly complex data necessary in the discovery phase of proteoform analysis. However, once we have in hand a comprehensive index of these proteoforms for the system under study, efforts can shift to a scoring mode informed by the previous knowledge. This transition from discovery to scoring is central to many fields: in genomics, for example, the initial discovery of single-nucleotide polymorphisms (SNPs) led to the generation of SNP databases and technologies for their scoring at scale. The scoring technology enabled cost-effective functional studies and disease-based research across human populations. Similarly, in MS, initial work to develop small molecule identification from gas-phase fragmentation patterns led to the establishment of rich databases of molecular fragmentation spectra allowing the rapid identification of already known compounds. This venerable principle will be invaluable to driving increased throughput and decreased cost. This anticipates that disruptive technologies such as single-molecule proteoform sequencing and analysis would benefit by providing a reference set of the human proteoforms actually present.

## ENABLING NEW LEVELS OF BIOMEDICAL RESEARCH

**Fig. 5.**
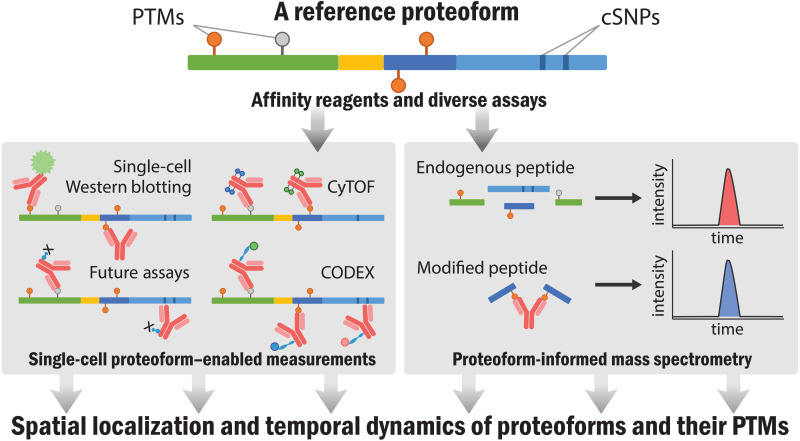
Once proteoforms have been identified, affinity reagents and targeted assays will enable emergent strategies to delineate their spatial distribution and temporal dynamics of proteoforms and their PTMs. CyTOF, mass cytometry; CODEX, CO-Detection by indEXing.

With a new generation of precision measurement tools, studies of mutations, disease, infection, and drug treatment will all operate with more detailed knowledge afforded by creation of a comprehensive proteoform index. This will further accelerate the goal of 21st century biomedicine such as regenerative biology, enhanced drug development, and better detection of human disease—all of which involve proteins. Beyond improving the use of proteins as biomarkers, the reference atlas of proteoforms will enable the study of their spatial and temporal distributions within cells and tissues, information presently impossible to obtain. This will often involve protein affinity capture reagents enabling readouts using a wide array of technologies (Fig. 5). Scoring technologies for single-molecule and single-cell biology will be propelled by having proteoform answers in the “back of the book” as we develop and optimize them in the decade ahead.

## SYNERGY OF THE HUMAN PROTEOFORM PROJECT WITH OTHER INITIATIVES

The Human Proteoform Project, by capturing all sources of protein variation for creation of a reference atlas of whole proteoforms, is fundamentally different from other proteomics initiatives. Prior initiatives such as those describing first drafts of the human proteome in 2014 (*33*, *34*) and ongoing work under the aegis of the Human Protein Atlas and the Human Proteome Project (*35*) have accomplished a great deal over the past several years, and the Human Proteome Organization has called for the community to “systematically map all human proteoforms” (*36*). There has also been an industry-led call from several pharmaceutical companies underscoring the need for major improvements in proteoform measurement (*37*).

Clear synergies with initiatives focused on human cell typing and protein capture reagents are visible. The Human Protein Atlas with its existing set of >15,000 antibodies provides an valuable resource for targeted studies while also driving efforts to develop “open source” renewable affinity reagents of known sequence (*38*). These affinity reagents enable targeted enrichment of proteoform families deriving from each human gene (Fig. 3, top). Once the members of proteoform families are known, creation of a next generation of proteoform-directed affinity reagents will be possible (Fig. 5) (*39*). An important thrust of the Human Proteoform Project is the delineation of proteoform expression patterns across human tissues and cell types to be archived in the Human Proteoform Atlas. This effort will benefit greatly from the output of the now accelerating efforts in the HuBMAP (*40*), the Human Cell Atlas (*41*), and several affiliated consortia. These groups are actively in the process of defining all human cell types in an organized and interoperable ontology. This includes generating markers of cell types that will facilitate their sampling for cell-based proteomics to determine the proteoforms present.

## ROLES OF GOVERNMENT, FOUNDATIONS, AND THE PRIVATE SECTOR

For the necessary transformation of technology and knowledge to take place over the coming decade, numerous stakeholders will be needed to engage and align with the project to bring it to fruition (*42*). Within the emergent proteomics ecosystem that we envisage, three categories of organizations can be identified—those focused on creating new knowledge (universities and research institutes), those creating new value for customers (instrument, biopharma, and diagnostics companies), and those providing financial and other resource support for the creation of knowledge or customer value (government agencies, philanthropies, nonprofit foundations, and well-established companies) (*43*). The role of the knowledge creators is paramount for a research-intensive area similar to this, and the major universities and research institutes will generate the structural, large-scale data to drive this effort. This will require substantial funding; for comparison, genomics research worldwide was publicly funded at about $3B per year from 2003 to 2006, with the United States contributing about 35% of this (*44*).

The companies and institutions that commercialize the tools, technologies, and services to advance the field also play a pivotal role in this endeavor often collaborating with academic researchers to bring new technologies to the marketplace. This cycle of innovation and commercialization was a fundamental enabler of the HGP. The biopharmaceutical and diagnostic companies invest heavily in research and development [for example, having spent $97 billion in R&D in the United States in 2017 (*45*)] and so are well poised to participate in these efforts. As noted above, generating the definitive proteoform set for the expressed human proteome presents a major economic opportunity for the private sector.

Bringing alignment and finding common goals for the various members of the emerging “proteoform ecosystem” is already underway with organizations starting to forge bridges across the boundaries. Increasing cooperation between public agencies, organizations, and international institutions will hasten the discovery and understanding of human proteoforms and provide marked growth in therapeutics, diagnostics, and the life sciences.

## CONCLUSION AND OUTLOOK

**Fig. 6.**
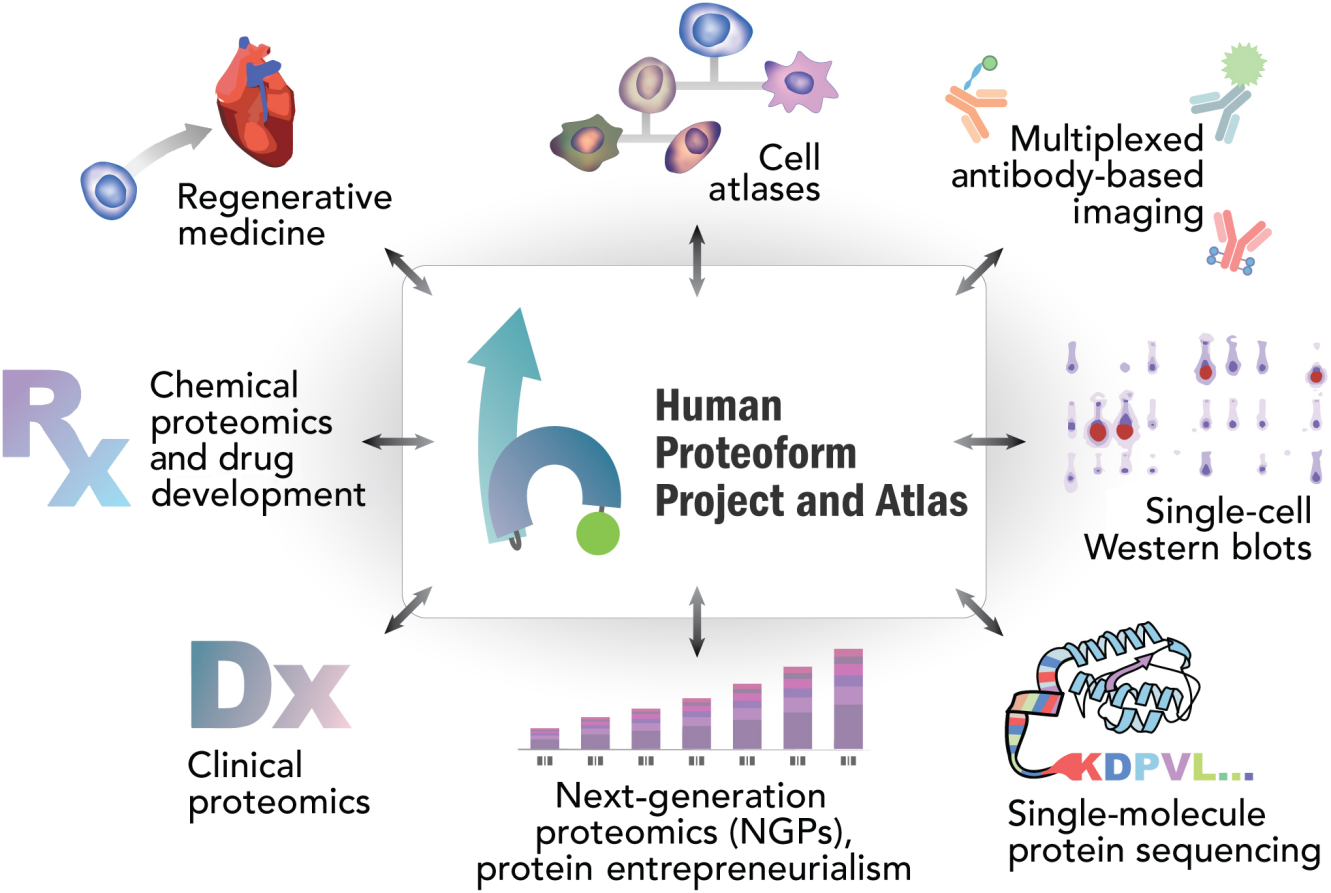
Projected interactions and impact from the Human Proteoform Project.

The Human Proteoform Project will revolutionize our understanding of human health and disease. This ambitious project to develop and apply powerful new technologies to reveal the molecular complexity that underlies human biology will be transformative. While a full exploration into the nature of its many impacts is beyond the scope of this article, we provide in Fig. 6 an overview of some of the many areas in which it will open new vistas and enable revolutionary new technologies. We offer the roadmap outlined here to inspire its realization.

## Supplementary Materials

### This PDF file includes:

Consortium for Top-Down Proteomics

## References

[1] N. Dranke, What is the human genome worth? Nature , (2011).

[2] F. S. Collins, E. D. Green, A. E. Guttmacher, M. S. Guyer; US National Human Genome Research Institute, A vision for the future of genomics research. Nature 422, 835–847 (2003). 12695777 10.1038/nature01626

[3] L. M. Smith, N. L. Kelleher; Consortium for Top Down Proteomics, Proteoform: A single term describing protein complexity. Nat. Methods 10, 186–187 (2013). 23443629 10.1038/nmeth.2369 PMC4114032

[4] R. Aebersold, J. N. Agar, I. J. Amster, M. S. Baker, C. R. Bertozzi, E. S. Boja, C. E. Costello, B. F. Cravatt, C. Fenselau, B. A. Garcia, Y. Ge, J. Gunawardena, R. C. Hendrickson, P. J. Hergenrother, C. G. Huber, A. R. Ivanov, O. N. Jensen, M. C. Jewett, N. L. Kelleher, L. L. Kiessling, N. J. Krogan, M. R. Larsen, J. A. Loo, R. R. Ogorzalek Loo, E. Lundberg, M. J. MacCoss, P. Mallick, V. K. Mootha, M. Mrksich, T. W. Muir, S. M. Patrie, J. J. Pesavento, S. J. Pitteri, H. Rodriguez, A. Saghatelian, W. Sandoval, H. Schlüter, S. Sechi, S. A. Slavoff, L. M. Smith, M. P. Snyder, P. M. Thomas, M. Uhlén, J. E. van Eyk, M. Vidal, D. R. Walt, F. M. White, E. R. Williams, T. Wohlschlager, V. H. Wysocki, N. A. Yates, N. L. Young, B. Zhang, How many human proteoforms are there? Nat. Chem. Biol. 14, 206–214 (2018). 29443976 10.1038/nchembio.2576 PMC5837046

[5] J. C. Venter, M. D. Adams, E. W. Myers, P. W. Li, R. J. Mural, G. G. Sutton, H. O. Smith, M. Yandell, C. A. Evans, R. A. Holt, J. D. Gocayne, P. Amanatides, R. M. Ballew, D. H. Huson, J. R. Wortman, Q. Zhang, C. D. Kodira, X. H. Zheng, L. Chen, M. Skupski, G. Subramanian, P. D. Thomas, J. Zhang, G. L. Gabor Miklos, C. Nelson, S. Broder, A. G. Clark, J. Nadeau, V. A. McKusick, N. Zinder, A. J. Levine, R. J. Roberts, M. Simon, C. Slayman, M. Hunkapiller, R. Bolanos, A. Delcher, I. Dew, D. Fasulo, M. Flanigan, L. Florea, A. Halpern, S. Hannenhalli, S. Kravitz, S. Levy, C. Mobarry, K. Reinert, K. Remington, J. Abu-Threideh, E. Beasley, K. Biddick, V. Bonazzi, R. Brandon, M. Cargill, I. Chandramouliswaran, R. Charlab, K. Chaturvedi, Z. Deng, V. D. Francesco, P. Dunn, K. Eilbeck, C. Evangelista, A. E. Gabrielian, W. Gan, W. Ge, F. Gong, Z. Gu, P. Guan, T. J. Heiman, M. E. Higgins, R. R. Ji, Z. Ke, K. A. Ketchum, Z. Lai, Y. Lei, Z. Li, J. Li, Y. Liang, X. Lin, F. Lu, G. V. Merkulov, N. Milshina, H. M. Moore, A. K. Naik, V. A. Narayan, B. Neelam, D. Nusskern, D. B. Rusch, S. Salzberg, W. Shao, B. Shue, J. Sun, Z. Y. Wang, A. Wang, X. Wang, J. Wang, M. H. Wei, R. Wides, C. Xiao, C. Yan, A. Yao, J. Ye, M. Zhan, W. Zhang, H. Zhang, Q. Zhao, L. Zheng, F. Zhong, W. Zhong, S. C. Zhu, S. Zhao, D. Gilbert, S. Baumhueter, G. Spier, C. Carter, A. Cravchik, T. Woodage, F. Ali, H. An, A. Awe, D. Baldwin, H. Baden, M. Barnstead, I. Barrow, K. Beeson, D. Busam, A. Carver, A. Center, M. L. Cheng, L. Curry, S. Danaher, L. Davenport, R. Desilets, S. Dietz, K. Dodson, L. Doup, S. Ferriera, N. Garg, A. Gluecksmann, B. Hart, J. Haynes, C. Haynes, C. Heiner, S. Hladun, D. Hostin, J. Houck, T. Howland, C. Ibegwam, J. Johnson, F. Kalush, L. Kline, S. Koduru, A. Love, F. Mann, D. May, S. McCawley, T. McIntosh, I. McMullen, M. Moy, L. Moy, B. Murphy, K. Nelson, C. Pfannkoch, E. Pratts, V. Puri, H. Qureshi, M. Reardon, R. Rodriguez, Y. H. Rogers, D. Romblad, B. Ruhfel, R. Scott, C. Sitter, M. Smallwood, E. Stewart, R. Strong, E. Suh, R. Thomas, N. N. Tint, S. Tse, C. Vech, G. Wang, J. Wetter, S. Williams, M. Williams, S. Windsor, E. Winn-Deen, K. Wolfe, J. Zaveri, K. Zaveri, J. F. Abril, R. Guigó, M. J. Campbell, K. V. Sjolander, B. Karlak, A. Kejariwal, H. Mi, B. Lazareva, T. Hatton, A. Narechania, K. Diemer, A. Muruganujan, N. Guo, S. Sato, V. Bafna, S. Istrail, R. Lippert, R. Schwartz, B. Walenz, S. Yooseph, D. Allen, A. Basu, J. Baxendale, L. Blick, M. Caminha, J. Carnes-Stine, P. Caulk, Y. H. Chiang, M. Coyne, C. Dahlke, A. D. Mays, M. Dombroski, M. Donnelly, D. Ely, S. Esparham, C. Fosler, H. Gire, S. Glanowski, K. Glasser, A. Glodek, M. Gorokhov, K. Graham, B. Gropman, M. Harris, J. Heil, S. Henderson, J. Hoover, D. Jennings, C. Jordan, J. Jordan, J. Kasha, L. Kagan, C. Kraft, A. Levitsky, M. Lewis, X. Liu, J. Lopez, D. Ma, W. Majoros, J. McDaniel, S. Murphy, M. Newman, T. Nguyen, N. Nguyen, M. Nodell, S. Pan, J. Peck, M. Peterson, W. Rowe, R. Sanders, J. Scott, M. Simpson, T. Smith, A. Sprague, T. Stockwell, R. Turner, E. Venter, M. Wang, M. Wen, D. Wu, M. Wu, A. Xia, A. Zandieh, X. Zhu, The sequence of the human genome. Science 291, 1304–1351 (2001). 11181995 10.1126/science.1058040

[6] L. M. Smith, N. L. Kelleher, Proteoforms as the next proteomics currency. Science 359, 1106–1107 (2018). 29590032 10.1126/science.aat1884 PMC5944612

[7] N. L. Kelleher, A cell-based approach to the human proteome project. J. Am. Soc. Mass Spectrom. 23, 1617–1624 (2012). 22976808 10.1007/s13361-012-0469-9 PMC3456959

[8] R. Aebersold, M. Mann, Mass-spectrometric exploration of proteome structure and function. 537, 347–355 (2016). 10.1038/nature19949 27629641

[9] Y. Zhang, B. R. Fonslow, B. Shan, M. C. Baek, J. R. Yates III, Protein analysis by shotgun/bottom-up proteomics. Chem. Rev. 113, 2343–2394 (2013). 23438204 10.1021/cr3003533 PMC3751594

[10] H. Zhang, T. Liu, Z. Zhang, S. H. Payne, B. Zhang, J. E. McDermott, J. Y. Zhou, V. A. Petyuk, L. Chen, D. Ray, S. Sun, F. Yang, L. Chen, J. Wang, P. Shah, S. W. Cha, P. Aiyetan, S. Woo, Y. Tian, M. A. Gritsenko, T. R. Clauss, C. Choi, M. E. Monroe, S. Thomas, S. Nie, C. Wu, R. J. Moore, K. H. Yu, D. L. Tabb, D. Fenyö, V. Bafna, Y. Wang, H. Rodriguez, E. S. Boja, T. Hiltke, R. C. Rivers, L. Sokoll, H. Zhu, I. M. Shih, L. Cope, A. Pandey, B. Zhang, M. P. Snyder, D. A. Levine, R. D. Smith, D. W. Chan, K. D. Rodland, S. A. Carr, M. A. Gillette, K. R. Klauser, E. Kuhn, D. R. Mani, P. Mertins, K. A. Ketchum, R. Thangudu, S. Cai, M. Oberti, A. G. Paulovich, J. R. Whiteaker, N. J. Edwards, P. B. McGarvey, S. Madhavan, P. Wang, D. W. Chan, A. Pandey, I. M. Shih, H. Zhang, Z. Zhang, H. Zhu, L. Cope, G. A. Whiteley, S. J. Skates, F. M. White, D. A. Levine, E. S. Boja, C. R. Kinsinger, T. Hiltke, M. Mesri, R. C. Rivers, H. Rodriguez, K. M. Shaw, S. E. Stein, D. Fenyo, T. Liu, J. E. McDermott, S. H. Payne, K. D. Rodland, R. D. Smith, P. Rudnick, M. Snyder, Y. Zhao, X. Chen, D. F. Ransohoff, A. N. Hoofnagle, D. C. Liebler, M. E. Sanders, Z. Shi, R. J. C. Slebos, D. L. Tabb, B. Zhang, L. J. Zimmerman, Y. Wang, S. R. Davies, L. Ding, M. J. C. Ellis, R. R. Townsend, Integrated proteogenomic characterization of human high-grade serous ovarian cancer. Cell 166, 755–765 (2016). 27372738 10.1016/j.cell.2016.05.069 PMC4967013

[11] P. Mertins, D. R. Mani, K. V. Ruggles, M. A. Gillette, K. R. Clauser, P. Wang, X. Wang, J. W. Qiao, S. Cao, F. Petralia, E. Kawaler, F. Mundt, K. Krug, Z. Tu, J. T. Lei, M. L. Gatza, M. Wilkerson, C. M. Perou, V. Yellapantula, K.-l. Huang, C. Lin, M. D. Mc Lellan, P. Yan, S. R. Davies, R. R. Townsend, S. J. Skates, J. Wang, B. Zhang, C. R. Kinsinger, M. Mesri, H. Rodriguez, L. Ding, A. G. Paulovich, D. Fenyö, M. J. Ellis, S. A. Carr; NCI CPTAC, Proteogenomics connects somatic mutations to signalling in breast cancer. Nature 534, 55–62 (2016). 27251275 10.1038/nature18003 PMC5102256

[12] S. Vasaikar, C. Huang, X. Wang, V. A. Petyuk, S. R. Savage, B. Wen, Y. Dou, Y. Zhang, Z. Shi, O. A. Arshad, M. A. Gritsenko, L. J. Zimmerman, J. E. M. Dermott, T. R. Clauss, R. J. Moore, R. Zhao, M. E. Monroe, Y.-T. Wang, M. C. Chambers, R. J. C. Slebos, K. S. Lau, Q. Mo, L. Ding, M. Ellis, M. Thiagarajan, C. R. Kinsinger, H. Rodriguez, R. D. Smith, K. D. Rodland, D. C. Liebler, T. Liu, B. Zhang; Clinical Proteomic Tumor Analysis Consortium, Proteogenomic analysis of human colon cancer reveals new therapeutic opportunities. Cell 177, 1035–1049.e19 (2019). 31031003 10.1016/j.cell.2019.03.030 PMC6768830

[13] K. V. Ruggles, Z. Tang, X. Wang, H. Grover, M. Askenazi, J. Teubl, S. Cao, M. D. McLellan, K. R. Clauser, D. L. Tabb, P. Mertins, R. Slebos, P. Erdmann-Gilmore, S. Li, H. P. Gunawardena, L. Xie, T. Liu, J. Y. Zhou, S. Sun, K. A. Hoadley, C. M. Perou, X. Chen, S. R. Davies, C. A. Maher, C. R. Kinsinger, K. D. Rodland, H. Zhang, Z. Zhang, L. Ding, R. R. Townsend, H. Rodriguez, D. Chan, R. D. Smith, D. C. Liebler, S. A. Carr, S. Payne, M. J. Ellis, D. Fenyő, An analysis of the sensitivity of proteogenomic mapping of somatic mutations and novel splicing events in cancer. Mol. Cell. Proteomics 15, 1060–1071 (2016). 26631509 10.1074/mcp.M115.056226 PMC4813688

[14] J. C. Tran, L. Zamdborg, D. R. Ahlf, J. E. Lee, A. D. Catherman, K. R. Durbin, J. D. Tipton, A. Vellaichamy, J. F. Kellie, M. Li, C. Wu, S. M. M. Sweet, B. P. Early, N. Siuti, R. D. LeDuc, P. D. Compton, P. M. Thomas, N. L. Kelleher, Mapping intact protein isoforms in discovery mode using top-down proteomics. Nature 480, 254–258 (2011). 22037311 10.1038/nature10575 PMC3237778

[15] B. Chen, K. A. Brown, Z. Lin, Y. Ge, Top-down proteomics: Ready for prime time? Anal. Chem. 90, 110–127 (2018). 29161012 10.1021/acs.analchem.7b04747 PMC6138622

[16] R. D. LeDuc, V. Schwämmle, M. R. Shortreed, A. J. Cesnik, S. K. Solntsev, J. B. Shaw, M. J. Martin, J. A. Vizcaino, E. Alpi, P. Danis, N. L. Kelleher, L. M. Smith, Y. Ge, J. N. Agar, J. Chamot-Rooke, J. A. Loo, L. Pasa-Tolic, Y. O. Tsybin, ProForma: A standard proteoform notation. J. Proteome Res. 17, 1321–1325 (2018). 29397739 10.1021/acs.jproteome.7b00851 PMC5837035

[17] L. M. Smith, P. M. Thomas, M. R. Shortreed, L. V. Schaffer, R. T. Fellers, R. D. LeDuc, T. Tucholski, Y. Ge, J. N. Agar, L. C. Anderson, J. Chamot-Rooke, J. Gault, J. A. Loo, L. Paša-Tolić, C. V. Robinson, H. Schlüter, Y. O. Tsybin, M. Vilaseca, J. A. Vizcaíno, P. O. Danis, N. L. Kelleher, A five-level classification system for proteoform identifications. Nat. Methods 16, 939–940 (2019). 31451767 10.1038/s41592-019-0573-x PMC6857706

[18] S. Dominguez-Medina, S. Fostner, M. Defoort, M. Sansa, A. K. Stark, M. A. Halim, E. Vernhes, M. Gely, G. Jourdan, T. Alava, P. Boulanger, C. Masselon, S. Hentz, Neutral mass spectrometry of virus capsids above 100 megadaltons with nanomechanical resonators. Science 362, 918–922 (2018). 30467165 10.1126/science.aat6457

[19] J. O. Kafader, R. D. Melani, K. R. Durbin, B. Ikwuagwu, B. P. Early, R. T. Fellers, S. C. Beu, V. Zabrouskov, A. A. Makarov, J. T. Maze, D. L. Shinholt, P. F. Yip, D. Tullman-Ercek, M. W. Senko, P. D. Compton, N. L. Kelleher, Multiplexed mass spectrometry of individual ions improves measurement of proteoforms and their complexes. Nat. Methods 17, 391–394 (2020). 32123391 10.1038/s41592-020-0764-5 PMC7131870

[20] J. O. Kafader, R. D. Melani, M. W. Senko, A. A. Makarov, N. L. Kelleher, P. D. Compton, Measurement of individual ions sharply increases the resolution of orbitrap mass spectra of proteins. Anal. Chem. 91, 2776–2783 (2019). 30609364 10.1021/acs.analchem.8b04519

[21] H. Ouldali, K. Sarthak, T. Ensslen, F. Piguet, P. Manivet, J. Pelta, J. C. Behrends, A. Aksimentiev, A. Oukhaled, Electrical recognition of the twenty proteinogenic amino acids using an aerolysin nanopore. Nat. Biotechnol. 38, 176–181 (2020). 31844293 10.1038/s41587-019-0345-2 PMC7008938

[22] L. Restrepo-Perez, C. Joo, C. Dekker, Paving the way to single-molecule protein sequencing. Nat. Nanotechnol. 13, 786–796 (2018). 30190617 10.1038/s41565-018-0236-6

[23] M. Beck, J. A. Malmström, V. Lange, A. Schmidt, E. W. Deutsch, R. Aebersold, Visual proteomics of the human pathogen Leptospira interrogans. Nat. Methods 6, 817–823 (2009). 19838170 10.1038/nmeth.1390 PMC2862215

[24] M. Xu, J. Singla, E. I. Tocheva, Y.-W. Chang, R. C. Stevens, G. J. Jensen, F. Alber, De novo structural pattern mining in cellular electron cryotomograms. Structure 27, 679–691.e14 (2019). 30744995 10.1016/j.str.2019.01.005 PMC7542605

[25] H. Specht, N. Slavov, Transformative opportunities for single-cell proteomics. J. Proteome Res. 17, 2565–2571 (2018). 29945450 10.1021/acs.jproteome.8b00257 PMC6089608

[26] B. Budnik, E. Levy, G. Harmange, N. Slavov, SCoPE-MS: Mass spectrometry of single mammalian cells quantifies proteome heterogeneity during cell differentiation. Genome Biol. 19, 161 (2018). 30343672 10.1186/s13059-018-1547-5 PMC6196420

[27] N. Slavov, Single-cell protein analysis by mass spectrometry. Curr. Opin. Chem. Biol. 60, 1–9 (2021). 32599342 10.1016/j.cbpa.2020.04.018 PMC7767890

[28] M. Zhou, N. Uwugiaren, S. M. Williams, R. J. Moore, R. Zhao, D. Goodlett, I. Dapic, L. Paša-Tolić, Y. Zhu, Sensitive top-down proteomics analysis of a low number of mammalian cells using a nanodroplet sample processing platform. Anal. Chem. 92, 7087–7095 (2020). 32374172 10.1021/acs.analchem.0c00467

[29] Y. Zhu, G. Clair, W. B. Chrisler, Y. Shen, R. Zhao, A. K. Shukla, R. J. Moore, R. S. Misra, G. S. Pryhuber, R. D. Smith, C. Ansong, R. T. Kelly, Proteomic analysis of single mammalian cells enabled by microfluidic nanodroplet sample preparation and ultrasensitive NanoLC-MS. Angew. Chem. Int. Ed. Eng. 57, 12370–12374 (2018). 10.1002/anie.201802843 PMC6261339 29797682

[30] J. Swaminathan, A. A. Boulgakov, E. T. Hernandez, A. M. Bardo, J. L. Bachman, J. Marotta, A. M. Johnson, E. V. Anslyn, E. M. Marcotte, Highly parallel single-molecule identification of proteins in zeptomole-scale mixtures. Nat. Biotechnol. 36, 1076–1082 (2018). 10.1038/nbt.4278 PMC6482110 30346938

[31] C. Wu, P. M. Garden, D. R. Walt, Ultrasensitive detection of attomolar protein concentrations by dropcast single molecule assays. J. Am. Chem. Soc. 142, 12314–12323 (2020). 32602703 10.1021/jacs.0c04331 PMC7368998

[32] L. Smith, J. Chamot-Rooke, P. Danis, Y. Ge, J. Loo, L. Pasa-Tolic, Y. Tsybin, N. Kelleher, The Human Proteoform Project: A plan to define the human proteome; www.preprints.org/manuscript/202010.0368/v1 (2020). 10.1126/sciadv.abk0734 PMC8589312 34767442

[33] M. S. Kim, S. M. Pinto, D. Getnet, R. S. Nirujogi, S. S. Manda, R. Chaerkady, A. K. Madugundu, D. S. Kelkar, R. Isserlin, S. Jain, J. K. Thomas, B. Muthusamy, P. Leal-Rojas, P. Kumar, N. A. Sahasrabuddhe, L. Balakrishnan, J. Advani, B. George, S. Renuse, L. D. N. Selvan, A. H. Patil, V. Nanjappa, A. Radhakrishnan, S. Prasad, T. Subbannayya, R. Raju, M. Kumar, S. K. Sreenivasamurthy, A. Marimuthu, G. J. Sathe, S. Chavan, K. K. Datta, Y. Subbannayya, A. Sahu, S. D. Yelamanchi, S. Jayaram, P. Rajagopalan, J. Sharma, K. R. Murthy, N. Syed, R. Goel, A. A. Khan, S. Ahmad, G. Dey, K. Mudgal, A. Chatterjee, T. C. Huang, J. Zhong, X. Wu, P. G. Shaw, D. Freed, M. S. Zahari, K. K. Mukherjee, S. Shankar, A. Mahadevan, H. Lam, C. J. Mitchell, S. K. Shankar, P. Satishchandra, J. T. Schroeder, R. Sirdeshmukh, A. Maitra, S. D. Leach, C. G. Drake, M. K. Halushka, T. S. K. Prasad, R. H. Hruban, C. L. Kerr, G. D. Bader, C. A. Iacobuzio-Donahue, H. Gowda, A. Pandey, A draft map of the human proteome. Nature 509, 575–581 (2014). 24870542 10.1038/nature13302 PMC4403737

[34] M. Wilhelm, J. Schlegl, H. Hahne, A. M. Gholami, M. Lieberenz, M. M. Savitski, E. Ziegler, L. Butzmann, S. Gessulat, H. Marx, T. Mathieson, S. Lemeer, K. Schnatbaum, U. Reimer, H. Wenschuh, M. Mollenhauer, J. Slotta-Huspenina, J. H. Boese, M. Bantscheff, A. Gerstmair, F. Faerber, B. Kuster, Mass-spectrometry-based draft of the human proteome. Nature 509, 582–587 (2014). 24870543 10.1038/nature13319

[35] P. J. Thul, L. Åkesson, M. Wiking, D. Mahdessian, A. Geladaki, H. A. Blal, T. Alm, A. Asplund, L. Björk, L. M. Breckels, A. Bäckström, F. Danielsson, L. Fagerberg, J. Fall, L. Gatto, C. Gnann, S. Hober, M. Hjelmare, F. Johansson, S. Lee, C. Lindskog, J. Mulder, C. M. Mulvey, P. Nilsson, P. Oksvold, J. Rockberg, R. Schutten, J. M. Schwenk, Å. Sivertsson, E. Sjöstedt, M. Skogs, C. Stadler, D. P. Sullivan, H. Tegel, C. Winsnes, C. Zhang, M. Zwahlen, A. Mardinoglu, F. Pontén, K. von Feilitzen, K. S. Lilley, M. Uhlén, E. Lundberg, A subcellular map of the human proteome. Science 356, eaal3321 (2017). 28495876 10.1126/science.aal3321

[36] S. Adhikari, E. C. Nice, E. W. Deutsch, L. Lane, G. S. Omenn, S. R. Pennington, Y. K. Paik, C. M. Overall, F. J. Corrales, I. M. Cristea, J. E. van Eyk, M. Uhlén, C. Lindskog, D. W. Chan, A. Bairoch, J. C. Waddington, J. L. Justice, J. LaBaer, H. Rodriguez, F. He, M. Kostrzewa, P. Ping, R. L. Gundry, P. Stewart, S. Srivastava, S. Srivastava, F. C. S. Nogueira, G. B. Domont, Y. Vandenbrouck, M. P. Y. Lam, S. Wennersten, J. A. Vizcaino, M. Wilkins, J. M. Schwenk, E. Lundberg, N. Bandeira, G. Marko-Varga, S. T. Weintraub, C. Pineau, U. Kusebauch, R. L. Moritz, S. B. Ahn, M. Palmblad, M. P. Snyder, R. Aebersold, M. S. Baker, A high-stringency blueprint of the human proteome. Nat. Commun. 11, 5301 (2020). 33067450 10.1038/s41467-020-19045-9 PMC7568584

[37] J. F. Kellie, J. C. Tran, W. Jian, B. Jones, J. T. Mehl, Y. Ge, J. Henion, K. P. Bateman, Intact protein mass spectrometry for therapeutic protein quantitation, pharmacokinetics, and biotransformation in preclinical and clinical studies: An industry perspective. J. Am. Soc. Mass Spectrom. 32, 1886–1900 (2021). 32869982 10.1021/jasms.0c00270

[38] M. Baker, Reproducibility crisis: Blame it on the antibodies. Nature 521, 274–276 (2015). 25993940 10.1038/521274a

[39] X. X. Zhou, C. J. Bracken, K. Zhang, J. Zhou, Y. Mou, L. Wang, Y. Cheng, K. K. Leung, J. A. Wells, Targeting phosphotyrosine in native proteins with conditional, bispecific antibody traps. J. Am. Chem. Soc. 142, 17703–17713 (2020). 32924468 10.1021/jacs.0c08458 PMC8168474

[40] HuBMAP Consortium, The human body at cellular resolution: The NIH Human Biomolecular Atlas Program. Nature 574, 187–192 (2019). 31597973 10.1038/s41586-019-1629-x PMC6800388

[41] A. Regev, S. A. Teichmann, E. S. Lander, I. Amit, C. Benoist, E. Birney, B. Bodenmiller, P. Campbell, P. Carninci, M. Clatworthy, H. Clevers, B. Deplancke, I. Dunham, J. Eberwine, R. Eils, W. Enard, A. Farmer, L. Fugger, B. Göttgens, N. Hacohen, M. Haniffa, M. Hemberg, S. Kim, P. Klenerman, A. Kriegstein, E. Lein, S. Linnarsson, E. Lundberg, J. Lundeberg, P. Majumder, J. C. Marioni, M. Merad, M. Mhlanga, M. Nawijn, M. Netea, G. Nolan, D. Pe'er, A. Phillipakis, C. P. Ponting, S. Quake, W. Reik, O. Rozenblatt-Rosen, J. Sanes, R. Satija, T. N. Schumacher, A. Shalek, E. Shapiro, P. Sharma, J. W. Shin, O. Stegle, M. Stratton, M. J. T. Stubbington, F. J. Theis, M. Uhlen, A. van Oudenaarden, A. Wagner, F. Watt, J. Weissman, B. Wold, R. Xavier, N. Yosef; Human Cell Atlas Meeting Participants, The human cell atlas. eLife 6, e27041 (2017). 29206104 10.7554/eLife.27041 PMC5762154

[42] R. Adner, Ecosystem as structure: An actionable construct for strategy. J. Manag. 43, 39–58 (2017).

[43] K. Valkokari, Business, innovation, and knowledge ecosystems: How they differ and how to survive and thrive within them. Technol. Innov. Manag. Rev. 5, 17–24 (2015).

[44] J. R. Pohlhaus, R. M. Cook-Deegan, Genomics research: World survey of public funding. BMC Genomics 9, 472 (2008). 18847466 10.1186/1471-2164-9-472 PMC2576262

[45] Biopharmaceutical Industry Profile (*PhRMA - Pharmaceutical Research and Manufacturers of America*, 2019).

[46] E. Dolgin, The most popular genes in the human genome. Nature 551, 427–431 (2017). 10.1038/d41586-017-07291-9 29168817

