# Supplementary material for: The Human Proteoform Project: Defining the human proteome
Source: Sci Adv. 2021 Nov 12;7(46):eabk0734. doi: 10.1126/sciadv.abk0734 (PMC8589312; doi:10.1126/sciadv.abk0734)
Supplement: Supplementary file 1 — Consortium for Top-Down Proteomics [file sciadv.abk0734_sm.pdf]

Science Advances
AAAS

Supplementary Materials for

The Human Proteoform Project: Defining the human proteome

Lloyd M. Smith, Jeffrey N. Agar, Julia Chamot-Rooke, Paul O. Danis,
Ying Ge, Joseph A. Loo, Ljiljana Paša-Tolić, Yury O. Tsybin, Neil L. Kelleher*,
The Consortium for Top-Down Proteomics

*Corresponding author: n-kelleher@northwestern.edu

Published 12 November 2021, *Sci. Adv.* **7**, eabk0734 (2021)
DOI: 10.1126/sciadv.abk0734

This PDF file includes:

Consortium for Top-Down Proteomics

Consortium for Top-Down Proteomics

- Paul O. Danis, Consortium for Top-Down Proteomics
    - Chief Executive Officer
- Neil L. Kelleher, Northwestern University
    - President, Board of Directors
- Lloyd M. Smith, University of Wisconsin
    - Treasurer, Board of Directors
- Ying Ge, University of Wisconsin
    - Secretary, Board of Directors
- Jeff N. Agar, Northeastern University
    - Board of Directors, Member At-Large
- Julia Chamot-Rooke, Institut Pasteur
    - Board of Directors, Member At-Large
- Joseph A. Loo, University of California, Los Angeles
    - Board of Directors, Member At-Large
- Ljiljana Paša-Tolić, Pacific Northwest National Laboratory
    - Board of Directors, Member At-Large
- Yury O. Tsybin, Spectroswiss Sàrl
    - Board of Directors, Member At-Large
